# Key Role of the Influenza A Virus PA Gene Segment in the Emergence of Pandemic Viruses

**DOI:** 10.3390/v12040365

**Published:** 2020-03-26

**Authors:** Michael M. Lutz, Megan M. Dunagan, Yuki Kurebayashi, Toru Takimoto

**Affiliations:** 1 Department of Microbiology and Immunology, University of Rochester Medical Center, Rochester, NY 14642, USAmegan_dunagan@urmc.rochester.edu (M.M.D.); yuki_kurebayashi@urmc.rochester.edu (Y.K.); 2 Department of Biochemistry, School of Pharmaceutical Sciences, University of Shizuoka, Shizuoka-shi 422-8526, Japan

**Keywords:** host adaptation, influenza A virus, PA, PA-X, RNA-dependent RNA polymerase

## Abstract

Influenza A viruses (IAVs) are a significant human pathogen that cause seasonal epidemics and occasional pandemics. Avian waterfowl are the natural reservoir of IAVs, but a wide range of species can serve as hosts. Most IAV strains are adapted to one host species and avian strains of IAV replicate poorly in most mammalian hosts. Importantly, IAV polymerases from avian strains function poorly in mammalian cells but host adaptive mutations can restore activity. The 2009 pandemic H1N1 (H1N1pdm09) virus acquired multiple mutations in the PA gene that activated polymerase activity in mammalian cells, even in the absence of previously identified host adaptive mutations in other polymerase genes. These mutations in PA localize within different regions of the protein suggesting multiple mechanisms exist to activate polymerase activity. Additionally, an immunomodulatory protein, PA-X, is expressed from the PA gene segment. PA-X expression is conserved amongst many IAV strains but activity varies between viruses specific for different hosts, suggesting that PA-X also plays a role in host adaptation. Here, we review the role of PA in the emergence of currently circulating H1N1pdm09 viruses and the most recent studies of host adaptive mutations in the PA gene that modulate polymerase activity and PA-X function.

## 1. Introduction

### 1.1. Influenza A Viruses

Influenza A viruses (IAVs) are members of the Orthomyxoviridae family of RNA viruses and are capable of infecting a wide array of species. IAV is classified into different subtypes dependent on two viral glycoproteins, hemagglutinin (HA) and neuraminidase (NA) [1]. Avian species such as waterfowl are the natural host reservoir of IAVs where 16 HA subtypes and 9 NA subtypes have been observed to circulate; in addition, two subtypes H17N10 and H18N11 have been observed in bats [2,3]. Currently only a few subtypes, H1N1 and H3N2, have maintained substantial transmission within the human population and are among the leading causes of respiratory infections annually. While seasonal epidemics caused by IAVs are a significant global health burden, IAVs also have the potential to cause pandemic outbreaks. Most strains of IAV are specifically adapted to one host species, but genomic reassortment between human and avian viruses has previously led to pandemic outbreaks. Pandemics occur when antigenically distinct viruses, to which the human population has no preexisting immunity, emerge and quickly spread. The natural avian reservoir of IAV is a major source of antigenically distinct viruses which can potentially cause pandemic outbreaks. Direct adaptation of avian IAVs to humans or the genomic assortment of human and avian IAV genes can lead to the introduction of these antigenically distinct viruses to the human population. Within the last century, four IAV pandemics have occurred: the H1N1 Spanish flu (1918), the H2N2 Asian flu (1957), the H3N2 Hong Kong flu (1968), and the H1N1 swine flu (2009). Genomic sequencing of the viruses behind these outbreaks has definitively linked avian IAVs to each pandemic. More recently, sporadic zoonotic infections in humans with highly pathogenic avian IAVs, such as H5N1 and H7N9, have been observed. While there is little evidence of human to human transmission, the adaptation of these antigenically novel viruses to humans is a mounting health concern as they may lead to the next pandemic outbreak. In order to cross the species barrier, these avian IAVs need to acquire adaptive mutations in certain viral genes [4]. Some of these genes include the viral glycoproteins HA and NA which mediate viral entry and release, respectively [4]. Additionally, activity of the viral RNA-dependent RNA polymerase (vRdRp) has been identified as a significant factor regulating the host adaptation of avian IAVs to mammals. 

### 1.2. The Influenza A Virus RNA-Dependent RNA Polymerase

The IAV genome is comprised of eight segmented, negative sense, single-stranded RNAs which are bound by nucleoprotein (NP) and the vRdRp [1]. Three subunits, PB1, PB2, and PA proteins, make up the vRdRp complex. The PB1 protein is the main catalytic unit of the vRdRp, responsible for transcribing viral mRNAs and replicating the viral genome (vRNA) through a positive sense intermediate (cRNA) [5]. PB2 and PA bind to PB1 to make up the core structure of the vRdRp which has been shown in a number of crystal structures, including a recently solved crystal structure of human H3N2 vRdRp [6]. It has been well described that vRdRp derived from avian IAVs do not function well in mammalian cells. However, it has also been determined that adaptive mutations in the polymerase subunits can rescue the functionality of avian vRdRp in mammalian cells [7]. The environment in which avian and human IAVs replicate differs significantly; the median temperature of the avian enteric tract is around 40 °C, while human airways range from 33 °C (upper respiratory tract) to 37 °C (lower respiratory tract). Temperature-sensitive adaptive mutations have been identified in the vRdRp and are even exploited when making live attenuated influenza vaccines [8]. Historically, the majority of host adaptive mutations that have been found in the influenza A vRdRp are located in the PB2 subunit. Very few adaptive mutations have been characterized in PB1, and in fact, avian PB1 is able to function in assays where mammalian-adapted PA and PB2 are present. This is supported by the fact that the 1957 and 1968 pandemic viruses contained an avian PB1 gene but PB2 and PA genes were from human adapted IAVs [9].

## 2. The Role of PA in Host Adaptation of the RNA-Dependent RNA Polymerase 

### 2.1. Host Adaptive Mutations in Polymerase Proteins

PB2 was the first subunit of the vRdRp that was found to harbor species-specific mutations that significantly affect polymerase activity [10]. The most well-studied host adaptive mutation in PB2 is at residue 627, which is typically a glutamic acid in avian IAVs and a lysine in mammalian IAVs [11]. A single mutation from glutamic acid to lysine at this position is capable of significantly activating avian vRdRp in mammalian cells. The PB2 E627K mutation is considered a hallmark residue for host adaptation of avian IAVs to mammals. The precise mechanism(s) by which this polymerase mutation activates avian vRdRp activity is not known. However, the most preferred mechanism that has long been hypothesized is the involvement of one or more species-specific cellular factor(s) in viral genome replication and/or transcription [12]. Since avian vRdRp is significantly restricted in its activity in mammalian cells, there are two possibilities for the involvement of a cellular factor. The first is that mammalian cells express antiviral restriction factors which inhibit avian vRdRp activities, thus adaptive mutations are required for escaping or overcoming the effect of the restriction factor(s). Alternatively, mammalian cells may lack a positive factor, or certain features of this factor, that is used by avian vRdRp in avian cells to ensure high polymerase activity. Interestingly, it was recently proposed that species-specific differences in the acidic nuclear phosphoproteins (ANPs), and specifically ANP 32 proteins, are involved in the restriction of avian vRdRp in mammalian cells, and the PB2 mutation, E627K, may play a role [13,14,15]. Another well-known mammalian adaptive mutation in PB2, D701N, has been shown to regulate interaction of PB2 with cellular importin-α proteins [16,17]. This mutation enhances the nuclear import of PB2 by disrupting a salt-bridge, making the nuclear localization signal in PB2 more accessible to host factors [18]. This enhanced nuclear import of PB2 is considered to affect viral transcription and replication, which takes place in the nucleus.

Recently, it has become apparent that the PA subunit can play a major role in the host adaptation of avian vRdRp. This was highlighted with the emergence of the H1N1pdm09 virus which originated from genomic reassortment events in swine. Genetic analysis determined that the PB1 gene segment was introduced to swine from a human IAV, while the PB2 and PA segments were recently introduced into swine from an avian IAV around 1998 [19]. What is interesting from the H1N1pdm09 virus is that the PB2 gene segment originated from an avian IAV and was maintained for approximately ten years in the swine population without acquiring previously described host-adaptive PB2 mutations such as E627K. Instead, our lab and others have found that the PA segment in the H1N1pdm09 virus contains a number of host-adaptive mutations that determine the activity of vRdRp in mammalian cells [20,21,22]. Additionally, there have been several studies regarding novel H5N1 and H7N9 avian IAVs whose replicative ability and virulence have been linked to mutations in PA in addition to the previously described host adaptive mutations in PB2 [23,24]. Together these studies show that the PA gene segment also plays a major role in the adaptation of avian IAVs to mammals and the emergence of pandemic viruses. 

### 2.2. Structural and Functional Domains of PA

**Figure 1:**
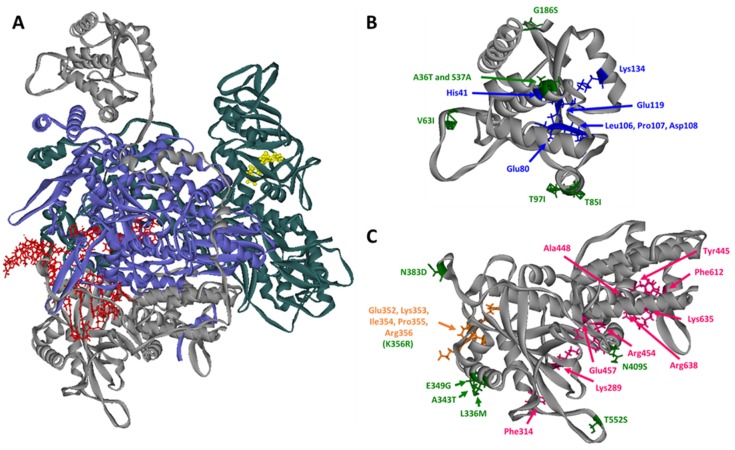
Structure of the Influenza A virus (IAV) polymerase and host adaptive mutations in PA. (**A**) Human H3N2 polymerase structure (PDB:6RR7) [6]. The PA subunit is colored gray, PB2 subunit is colored teal, and PB1 subunit is colored blue. Viral genome (vRNA) bound to the PB1 and PA subunits is colored red and a 5′ 7-methylguanosine (m7G)-capped substrate bound to PB2 is colored yellow. (**B**) Detailed view of the PA endonuclease domain with host adaptive mutations is indicated in green and residues involved in endonuclease activity are indicated in blue. (**C**) Detailed view of the PA C-terminal domain with host adaptive mutations indicated in green, residues involved in binding to RNA Pol II C-terminal domain peptides in pink, and residues involved in dimerization in orange.

Within the viral polymerase complex, the PA protein is the smallest subunit at 716 amino acids in length [25]. The PA protein is encoded for by segment 3 of IAV and can essentially be divided into two major domains: the N-terminal domain which is approximately the first 200 amino acids and the C-terminal domain which includes amino acids 255–716 [26]. These two domains are on opposite sides of the vRdRp complex and are connected by a flexible amino acid linker region. A crystal structure of the human H3N2 vRdRp and the two major domains of PA are detailed in Figure 1 [6]. Recent structural and biochemical studies have provided considerable new information about the vRdRp and PA. In 2009, the crystal structure of the N-terminal region of PA was solved by two independent groups [27,28]. This exciting work revealed that the N-terminal domain of PA contains an endonuclease active site motif, similar to type II restriction endonucleases [29]. Another major step forward was made in 2017 when the crystal structure of an H17N10 bat vRdRp was solved in complex with an RNA pol II C-terminal domain (CTD) mimetic peptide [30]. More recently in 2019, crystal structures of human H3N2 and avian H5N1 vRdRp were obtained [6]. These crystal structures show a high level of similarity to the H17N10 bat vRdRp and show dimerization of the polymerase. These studies, along with fundamental biochemical characterizations of polymerase activity have significantly enhanced our understanding of IAV host adaptation. Despite having an unknown function for many years, the multiple essential functions of PA in the vRdRp complex and viral life cycle are becoming clearer. 

As part of the vRdRp, PA helps to form the core structure of the polymerase with PB1 and PB2 [31,32]. Functional studies have also shown that the N-terminal domain of PA contains regions which are involved in binding to the vRNA/cRNA promoter [33]. Most importantly, the endonuclease active site in PA plays an essential role in cap-snatching, which is required for viral mRNA transcription. Cap-snatching is the process by which the vRdRp acquires 5′ 7-methylguanosine (m7G) caps for viral mRNA [34]. It has long been appreciated that viruses have very diverse ways of initiating translation by host cell machinery. These include internal ribosome entry sites which are inherent structures in the 5′ untranslated region (UTR) of viral mRNAs that recruit translation factors and/or ribosomes [35]. Additionally, many viruses take advantage of the canonical cap-dependent translation initiation process that is utilized by the host cell and is dependent on the presence of a 5′ m7G cap structure which recruits translation factors and ribosomes. Some viruses encode their own proteins which add the 5′ m7G cap structure onto viral mRNAs and others utilize cellular proteins to add the cap structure [36]. However, IAV was the first virus discovered to steal 5′ m7G caps from nascent transcribed host RNAs [37]. This is mediated by the vRdRp binding to cellular RNA polymerase II (Pol II) to gain access to host RNAs which have recently been capped [38]. The PB2 subunit binds to the 5′ m7G cap structure and the endonuclease domain of PA cleaves the host RNA several nucleotides downstream of the 5′ cap [27,28,39]. This creates a short capped primer which is then inserted into the PB1 active site and used to initiate viral mRNA transcription from the vRNA genomic template [26]. This unique mechanism of transcription initiation means that viral mRNAs are actually chimeras of host RNAs and viral mRNA.

PA is believed to contain two areas involved in nuclear localization in the N-terminal domain [40]; although PA by itself is not efficiently imported into the nucleus and instead relies on an interaction with PB1 for efficient nuclear import [41,42]. In contrast to the N-terminal domain, the C-terminal domain of PA was thought to mostly be involved in interactions with PB1 to form the core structure of the vRdRp [31,32]. However, several recent studies have highlighted the importance of the C-terminal domain of PA in regulating vRdRp activities. Two binding sites for the Ser5 phosphorylated C-terminal domain of RNA Pol II have been found to be located in the C-terminal domain of PA [30]. Other structural data of the vRdRp have shown that there are two distinct forms: a “transcriptionally inactive” form and “transcription preinitiation” form [43,44,45]. A model has been suggested for the influenza C vRdRp in which binding of the RNA pol II C-terminal domain to P3 (equivalent to PA in IAV) regulates the conformational change between these two forms of the vRdRp by stabilizing the “transcription preinitiation” form [46]. While the mechanism for conformational regulation of the influenza A vRdRp still remains unclear, it is very likely that the binding of the RNA pol II C-terminal domain to PA plays an important role. Dimerization of the influenza A vRdRp has also been recently shown and is mediated by interactions between the C-terminal domain of PA and a small portion of the N-terminal domain of PB2 [6]. 

### 2.3. Contribution of Mutations in PA to Host Adaptation

While mutations in PB2 have long been known to regulate host adaptation, recent studies indicate that PA mutations also play a major role [7,47]. Table 1 highlights experimentally-verified host adaptive mutations in PA that are involved in avian to mammalian transmission and the locations of these mutations are shown in Figure 1. Many of these host adaptive mutations in PA were not identified until after the outbreak of the H1N1pdm09 virus. As previously mentioned, this virus did not contain typical mammalian adaptive mutations in PB2 and instead mutations in PA were found to play a larger role in modulating polymerase activity [20,21]. Additionally, the recent outbreaks of highly pathogenic avian IAVs led to identification of previously unknown host adaptive mutations in PA. Characterization of H5N1 viruses, which have caused severe disease in humans, shows that some of these viruses contain the characteristic mutations E627K and D701N in PB2 [23]. However, detailed analysis of H5N1 viruses also revealed the presence of host adaptive mutations in PA. In particular, this includes mutations at residues A343T and N383D that activate polymerase activity in human cells in the absence of PB2 E627K or D701N [24,48]. In addition to these experimentally confirmed adaptive mutations in PA, some bioinformatics analysis suggest that there are additional residues involved in mammalian host adaptation [49,50,51,52]. However, many of these residues still need to be experimentally validated to be certain they do in fact play a role in host adaptation. Experimentally validating these residues involves characterizing how they would affect viral mRNA transcription, viral RNA replication, and overall viral polymerase activity, alongside viral growth and pathogenicity in mammalian hosts.

### 2.4. Potential Mechanisms by which Host Adaptive Mutations in PA Modulate Polymerase Activity

Despite being able to identify a significant number of mutations in PA that contribute to the host adaptation of IAV, the mechanism(s) by which these mutations modulate viral replication and transcription in mammalian hosts are largely unknown. So far, no cellular factors have been identified that determine host specificity through their interactions with PA. However, there are a number of host proteins reported to interact with PA in addition to RNA Pol II. Several large proteomics studies were carried out by various investigators to identify the IAV interactome [53,54,55,56,57,58,59]. While these studies identified hundreds of potential host proteins that interact with PA, very few of these were independently verified. Additionally, the varied conditions under which these proteomics experiments were carried out complicates matters, as very few observed interactions between PA and host proteins overlap between these studies. Despite this, there is a handful of proteins individually verified to interact with PA including HAX1, CLE, and UBA52. HAX1 is suggested to be involved in the nuclear translocation of PA which is interesting because previous reports have shown that despite PA having two likely nuclear localization sequences in its N-terminus, it is not efficiently imported into the nucleus by itself. Reportedly, HAX1 binds PA and occludes the nuclear localization signals, but the presence of PB1 reduces HAX1 binding to PA, allowing the PA-PB1 heterodimer to be imported into the nucleus [60]. The other two proteins of note, UBA52 and CLE, appear to be involved in viral replication by as of yet undefined mechanisms [59,61]. CLE was also detected in purified IAV virions where it may be bound to viral ribonucleoprotein complexes [62]. It is unclear whether the presence of host adaptive mutations in PA modulates its interaction with any of these host factors.

Many of the identified host adaptive mutations in PA are located within the N-terminus, which contains the endonuclease domain, thus raising the possibility that cap-snatching is affected by these mutations and plays an important role in host adaptation. Recent advances in next generation sequencing (NGS) technology have now provided more information than ever before on the cap-snatching process. Several studies have analyzed the host-derived cap-snatched sequences on the 5′ UTR of IAV mRNAs to determine the specificity of target host RNAs for IAV cap-snatching [63,64]. One of the most interesting and unexpected findings was that non-coding RNAs are targeted for cap-snatching and comprise the majority of snatched primers [65]. This is contrary to what was previously believed, as it was thought that cap-snatching and subsequent degradation of host mRNAs by the vRdRp were additional measures of inhibiting of host translation. 

Detailed deep sequencing analyses of IAV mRNAs found that cap-snatching most likely does not target specific RNAs but rather occurs based on the basal rate of host RNA transcription by RNA Pol II. Non-coding RNAs such as the U1 and U2 small nuclear RNAs have high rates of transcription and as such appear as some of the most frequently cap-snatched transcripts [71]. These studies also defined the precise conditions for cap-snatching. The median length for cap-snatched primers is 11 or 12 nucleotides depending on the influenza vRNA template, and the endonuclease activity of PA preferentially cleaves after a 3′ G. Deep sequencing the cap-snatched primers from influenza mRNAs also provided definitive evidence for a mechanism to rescue suboptimal capped primers that are snatched by the vRdRp [72]. While the median length of cap-snatched primers that are attached to an influenza mRNA is 11 or 12 nucleotides, not all primers that are snatched are initially the appropriate length. About 15–20% of primers which are snatched from host RNAs go through a process termed prime and realignment (PAR), which is most likely related to the primer size when it is snatched [73]. Like cap-snatching, PAR is a process that has been described for negative sense, segmented, single-strand RNA viruses [72,74]. After a primer is snatched, it can be further processed by the vRdRp in which additional nucleotides are added to the 3′ end of the primer. It is assumed that these additional nucleotides are added because the original primer was not of sufficient length or complementarity for the viral RNA template. For IAVs, the length of the primer inversely correlates with the probability of PAR occurring, with primers shorter than nine nucleotides undergoing PAR at a rate of 73.6% [73]. Due to the structural relationship between the PA endonuclease domain, the PB1 catalytic site, the RNA pol II C-terminal domain binding pocket, and the PB2 cap-binding domain, it is very likely that mutations in the vRdRp affect host–protein interactions involved in the cap-snatching process.

Another possible mechanism of how host adaptive mutations in PA affect vRdRp activity has to do with the interaction between PA and RNA pol II. Crystal structures show that two binding pockets on the C-terminal domain of PA are positively charged to allow for the specificity of the vRdRp binding to the Ser5 phosphorylated form of RNA pol II [30]. This Ser5 phosphorylation on the C-terminal domain of RNA pol II is critical because it regulates the capping of nascent host RNAs; as transcription proceeds, Ser5 is gradually dephosphorylated while the transcript elongates [75,76]. The key residues in these two binding pockets on PA (S289, R454, K635, and R638) are highly conserved amongst different IAVs, but there are multiple host adaptive mutations around these sites as shown in Figure 1 and Table 1 [30]. Most interesting is the host adaptive mutation T552S in PA; in avian IAVs T552 is prominent while S552 is highly conserved in human adapted IAVs [21]. The crystal structure of H17N10 bat IAV polymerase in complex with an RNA pol II mimetic peptide shows that residue 547 (corresponds to residue 552 in human and avian IAVs) is located within the second binding pocket and very close to RNA pol II CTD residues [30]. While it has yet to be experimentally verified, it will be interesting if interactions with the RNA pol II C-terminal domain affect host specificity of the vRdRp [77,78,79].

Lastly, host adaptive mutations may affect the regulation of transcription and genome replication in specific hosts. Several models have been proposed over the years for the mechanism that regulates the switch from viral mRNA transcription to cRNA transcription and subsequent vRNA replication. A detailed analysis of viral transcription and replication indicates that initial transcripts produced from infecting IAV virions can be detected rapidly following infection. Viral mRNA can be detected almost immediately following infection while cRNA can be detected within four hours and further genome replication to produce both vRNA and cRNA can continue thereafter [80]. Some studies suggest that the non-structural nuclear export protein (NEP/NS2) or small viral RNAs play a role in regulating the switch from viral transcription to genome replication by directly interacting with the vRdRp [81,82]. However, it was also found that in vitro, the presence of high concentrations of additional copies of the vRdRp was able to trigger the switch from mRNA transcription to replication [83]. This is somewhat in agreement with this recent structural data which suggest that the dimerization of two vRdRps is required for the initiation of vRNA synthesis from a cRNA template and that this dimerization interface involves the C-terminal domain of PA and N-terminal domain of PB2 [6]. The residues in PA that are involved in this dimerization are 352–356 and a number of host-adaptive mutations have been observed in this area including 336M, 349G, and 356R [20,69,70]. The host adaptive mutation, K356R, is one of the residues identified to be involved in dimerization. This mutation, which was characterized in an avian H9N2 virus, increases vRdRp activity, viral replication in cell culture, and pathogenicity in a mouse model. How this mutation may contribute to dimerization of the vRdRp remains to be seen [70]. It is possible that the dimerization of the polymerase could be regulated in a species-specific manner and be related to host-adaptation [84]. Future studies will be necessary to test the effect of these and other previously identified mutations on dimerization and the switch from viral mRNA transcription to viral genome replication. 

### 2.5. Additional Proteins Expressed from the PA Gene Segment

Apart from PA, three additional proteins are expressed from the PA gene segment. PA-N182 and PA-N155, which were discovered in 2013, arise from translation initiation at the 11th and 13th internal start codons, respectively [85]. These two proteins lack the N-terminal region of PA but share the C-terminal region. Little is known about their function or role in the viral life cycle, however the start codons for PA-N182 and PA-N155 are highly conserved amongst different strains of IAV. While loss of these proteins appears to have no effect on overall vRdR activity, as determined by reporter gene assays, a study showed that loss of PA-N155 affects viral replication and pathogenicity in a mouse model [85]. 

In contrast, the accessory protein PA-X is much better characterized due to its major function in virus-induced host shutoff activity. PA-X was discovered to be expressed from PA mRNA by a ribosomal frameshifting event in 2012 [86]. During translation ribosomes stall at a U-rich stretch in the PA mRNA following a slow-to-decode codon and a small fraction of ribosomes skip forward one nucleotide [87]. This rare +1 ribosomal frameshift event is estimated to occur for less than 2% of translation events and results in translation of the novel protein PA-X that shares the first 191 amino acids with PA while acquiring a unique C-terminus consisting of either 41 or 61 amino acids [86,87]. The length of this unique PA-X C-terminus can vary depending on the strain and is commonly referred to as the X open reading frame (X-ORF). The frameshift motif is highly conserved amongst IAVs, suggesting that PA-X is essential for the viral life cycle [88].

## 3. The Role of PA-X in Host Adaptation of Influenza A Virus

### 3.1. Host Shutoff Activity of Influenza A Virus 

IAV has multiple mechanisms that target host gene expression profiles such as RNA synthesis, stability, and translation [89,90,91]. The culmination of these targeted mechanisms results in an overall reduction of host mRNA levels which is matched by decreased host protein translation [92]. This process, termed “host shutoff”, is a classical strategy utilized by many viruses [93,94]. Early studies have shown that IAV most significantly achieves host shutoff through the degradation of host mRNAs [91]. This degradation of host mRNAs was thought to be a byproduct of the cap-snatching process since the first identified targets of cap-snatching were host mRNAs and it was not until very recently that other capped RNAs were discovered to also be cap-snatched [25,27,28,34]. While cleavage of the 5′ cap from host RNAs leaves them more susceptible to degradation, IAV has more specific mechanisms for targeting host mRNAs. 

The first described host shutoff factor of IAV was the non-structural protein 1 (NS1), which has multiple functions [95,96]. NS1 is perhaps best known for its role in antagonizing cellular interferon (IFN) responses by blocking retinoic acid-inducible gene I (RIG-I) activation and preventing nuclear localization of transcription factors that regulate expression of anti-viral proteins [96,97]. NS1 from human IAVs also suppresses host protein synthesis by preventing maturation of host transcripts [98,99]. In this context, NS1 has been shown to bind the cleavage and polyadenylation specificity factor 30 (CPSF30), a 30 kDa subunit of the CPSF complex which is involved in the cleavage and maturation of the 3’ signaling region of newly synthesized cellular pre-mRNAs [98]. Crystal structures have shown that two NS1 molecules and two CPSF30 molecules form a complex and NS1 residues F103, M106, K108, D125, and D189 play a critical role in the interaction with CPSF30 [100]. Typically, these key NS1 residues are highly conserved amongst human IAVs. Interestingly, NS1 from the H1N1pdm09 virus has mutations in several of these conserved residues (K108R, D125E, and D189G), and therefore is inactive and unable to induce general host shutoff by blocking pre-mRNA maturation [101].

Although only discovered recently, the impact of PA-X on IAV-induced host shutoff is much greater than that of NS1. Studies in our lab using recombinant H1N1pdm09 viruses expressing active or inactive NS1 (on the basis of mutations in the conserved CPSF30 binding residues) or PA-X clearly indicate that PA-X is the major driving force in general host shutoff [102]. While the frameshift motif leading to PA-X expression is highly conserved amongst different strains of IAVs, the shutoff activity of PA-X is variable. Some studies, including ours, suggest that PA-X from human-adapted IAVs is less active than that of PA-X from avian-adapted IAVs. In agreement with this, H1N1pdm09 viruses expressed a PA-X protein with high shutoff activity likely due to their avian-origin PA gene [19,101,103,104]. The difference in shutoff activity between avian and human PA-X proteins is possibly due to mutations within the N-terminal domain that is shared with PA [103,105]. Recent transcriptomic analysis indicates that a virus expressing shutoff active NS1 with reduced PA-X expression most efficiently suppresses antiviral and innate immune responses in human cells. Interestingly, it appears that the mRNA from the NS gene segment can also become a target of PA-X mediated degradation in infected cells [102]. These studies suggests that IAV needs to finely tune the activities of these two shutoff proteins in order to circumvent host responses for optimum viral replication and transmission.

### 3.2. Structural and Functional Domains of PA-X

**Figure 2:**
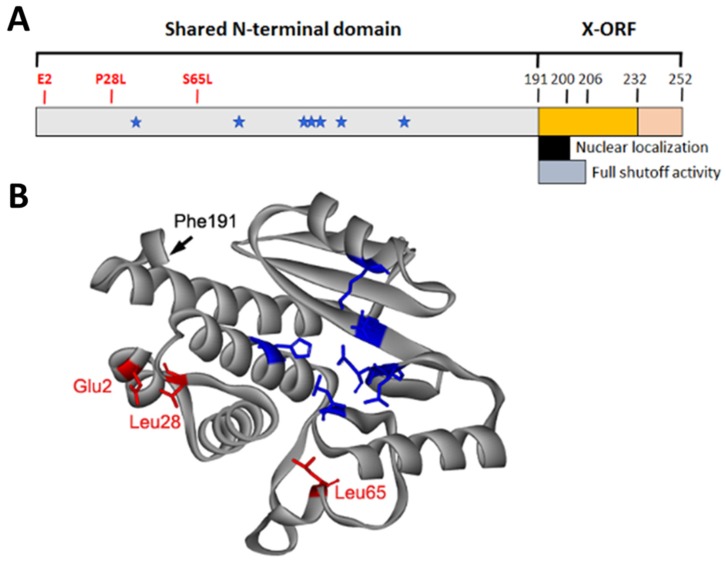
Structure of the IAV PA-X protein. (**A**) Schematic diagram of PA-X showing functional domains. Residues within the shared N-terminal domain known to affect PA-X shutoff activity are shown in red. Blue stars indicate the location of residues involved in endonuclease activity as previously shown in Figure 1. (**B**) Crystal structure of H3N2 PA N-terminal domain (PDB: 2W69) showing the location of key residues highlighted in (A) and the beginning of the X open reading frame (X-ORF) at Phe 191 [27].

Unlike NS1 which is proposed to prevent mRNA processing through its interaction with CPSF30, PA-X directly degrades host mRNAs through its N-terminal endonuclease domain. In fact, it has been shown that mutations in the endonuclease domain are able to completely inactivate PA-X shutoff activity [103,106,107]. In an effort to characterize PA-X functionality, several groups have identified amino acid residues which are critical for PA-X shutoff activity. Figure 2 shows the functional domains of PA-X and the location of amino acid residues known to affect shutoff activity on a crystal structure of the PA N-terminal domain. Initially, the X-ORF was the subject of intense study as it contains a unique sequence not found in the full-length PA protein that could explain the distinct functions of PA and PA-X. Sequence analysis identified two strain-dependent isoforms of PA-X which have either a 61 or 41 amino acid X-ORF. These two isoforms are hypothesized to play a role in host adaptation, as avian origin PA-X typically contains the 41 amino acid X-ORF while the X-ORF in human IAVs is typically 61 amino acids in length [86,88]. The additional C-terminal 20 residues in the 61 amino acid X-ORF do not appear to have a significant impact on PA-X shutoff activity, as determined by reporter gene assays [104,108]. However, one study showed that viruses containing PA-X with the 61 amino acid X-ORF exhibited enhanced viral replication and virulence in mice compared to viruses expressing the 41 amino acid X-ORF, suggesting that the C-terminal 20 residues have an additional function to enhance viral replication in vivo [109]. As of yet, the current body of literature does not fully address the function of these additional 20 C-terminal residues.

Apart from the differences in overall length of the X-ORF, questions arose about the existence of functional domains within the X-ORF. Several studies have demonstrated the importance of the first 15 amino acids of the X-ORF in regulating overall PA-X shutoff activity. Truncated PA-X constructs containing only the first 15 amino acids from the X-ORF display shutoff activity similar to full-length PA-X containing either the 41 or 61 amino acid X-ORF. Furthermore, loss of all X-ORF residues significantly inactivates PA-X shutoff activity, showing the critical role that these first 15 X-ORF residues play in host shutoff [104,108]. Quantitative subcellular fractionation revealed that PA-X localizes equally in both the cytoplasm and nucleus, and the N-terminal nine amino acids in the X-ORF are required for nuclear localization [104]. Although it has been shown that host RNA transcription is not required for RNA degradation by PA-X, mutations that decrease or prevent nuclear localization of PA-X greatly reduce shutoff activity [104,107,110]. Despite the dual localization of PA-X, the reduced functionality when excluded from the nucleus suggests a significant portion of its activity is derived through its nuclear localization. Furthermore, re-localization of a truncated PA-X lacking the X-ORF to the nucleus by addition of an exogenous SV40 nuclear localization signal can partially restore shutoff activity. However, the failure to restore full shutoff activity suggests that the first 15 amino acids of the X-ORF play a role in both nuclear localization and functionality [104,111].

Various groups have also determined the role of the shared N-terminal domain in regulating PA-X activity. As previously mentioned, key residues in the endonuclease active site are essential for PA-X activity as shown in Figure 1 and Figure 2. Apart from these residues in the catalytic site, reporter gene assays have been used to identify critical residues for PA-X activity. An initial study using chimeric PA-X proteins constructed from highly active PA-X (A/California/04/2009 (H1N1)) and weakly active PA-X (A/WSN/33 (H1N1)) showed that multiple residues within the N-terminal domain affect PA-X shutoff activity [103]. Recently, a similar approach using PA-X derived from the same two strains identified two key residues at 28 and 65 that affect PA-X shutoff activity significantly [105]. The importance of these residues was confirmed in other viral strains including A/Yokohama/UT2017/2003 (H3N2), A/Vietnam/HN31604/2009 (H5N1), and A/Anui/1/2013 (H7N9) [110]. These data also suggest that specific residues regulate overall PA-X shutoff activity in a species-specific manner, as P28 and S65 correlate to stronger PA-X activity and are predominantly found in avian origin IAVs. In the context of the overall structure of PA-X, residues 28 (located in helix alpha 4) and 65 (located in the flexible loop region) are far from the endonuclease active site. However, mutations in residue 65 have previously been characterized to change the conformation of the flexible loop region [27,112]. Taken together, these data suggest that PA-X adjusts its shutoff activity through mutations at residues far from the endonuclease active site and may play a role in modulating interactions with cellular factors. 

Additionally, a recent study reported a posttranslational modification on the N-terminal E2 residue of PA-X by an acetyltransferase, NatB [113]. Acetylation of this residue was found to stimulate PA-X shutoff activity, although the mechanism is still not fully understood. The effects of acetylation on individual proteins is hard to predict, with N-terminal acetylation reported to differentially effect protein–protein interactions, localization, stability, and structure of various cellular proteins [114,115]. However, acetylation of E2 in PA was also shown to be required for the function of PA as a component of vRdRp, highlighting the importance of posttranslational modifications in PA or PA-X [113]. Further work is needed to understand how exactly this post translational modification affects PA-X shutoff activity. Interestingly, in the structure of the N-terminal of PA-X, residues 2 and 28 are in close proximity to each other and the beginning of the X-ORF appears to fold back near this N-terminal region, suggesting that this region forms a critical domain required for the shutoff function of PA-X. Taken together, these data suggest that coordination between the N-terminal region and X-ORF is essential for PA-X activity, possibly through association with key host factors.

### 3.3. Mechanism of Host mRNA Degradation by PA-X

It has been shown that PA-X selectively targets cellular RNA pol II transcripts in the nucleus while sparing products of pol I and pol III [107]. While this explains specificity for targeting host mRNAs, as viral mRNAs are transcribed by viral RdRp, the mechanism of how host pol II transcripts are targeted by PA-X is not fully elucidated. Unlike the full-length PA in the vRdRp complex, PA-X lacks the C-terminal domain of PA which allows for specific interactions with the Ser5 phosphorylated RNA pol II C-terminal domain; therefore an additional mechanism must exist to allow for PA-X specificity for RNA pol II transcripts. One possibility is that PA-X interacts with cellular factors involved in pol II transcription or transcript processing. In agreement with this, a recent study indicated that PA-X preferentially degrades spliced pol II transcripts [111]. Additionally, a proteomic analysis using both the 61 and 41 amino acid X-ORFs from PA-X suggested there are a number of PA-X interactions with cellular proteins involved in mRNA processing. This analysis revealed 156 candidate proteins including 29 high confidence interactions that can be divided into four main groups: RNA splicing factors, pre-mRNA processing factors, proteasome factors, and proteins involved in nuclear import/export. Interaction of PA-X with NUDT21, a component of the CFIm complex that modulates polyadenylation of pre-mRNA, was confirmed by co-immunoprecipitation [111]. Based on these data a model was proposed whereby PA-X associates with a discrete set of RNA metabolism proteins that allows selective targeting of pol II transcripts during transcription or early processing [111]. In addition to interactions through the X-ORF, PA-X likely interacts with other host factors through the N-terminal domain as well. Future studies to unravel additional interacting partners and potential differences amongst various IAV strains are needed to fully understand PA-X induced host shutoff activity and its potential role in host adaptation.

### 3.4. Impact of PA-X on Influenza A Virus Pathogenicity 

In addition to mutations that enhance vRdRp activity, the modulation of host gene expression, especially related to the innate and acquired immune responses, is critical for the success of IAV infection and host adaptation. There is an obvious interest in the role PA-X plays in IAV virulence and pathogenesis because of the impact of host cytokine responses and inflammation on viral pathogenicity [116]. Studies using PA-X frame shift mutant viruses that express limited amounts of PA-X in avian origin H5N1 or H1N1pdm09 viruses have shown that PA-X downregulates interferons (IFNs) and pro-inflammatory cytokines that are produced early in infection [86,106,117]. Additionally, reduced expression of PA-X in the 1918 H1N1 and H1N1pdm09 viruses increased pathogenicity in a mouse model [86,106,117]. This phenomenon is also supported by experiments utilizing avian H5N1 strains, where loss of PA-X expression increased IAV virulence in mice, chickens, and ducks [118]. Similar results were observed for swine origin viruses, where expression of PA-X was shown to decrease virulence in a mouse model [119]. Overall these studies suggest that expression of PA-X decreases viral pathogenicity, highlighting the impact of host immune-induced pathology during IAV infection [118,120]. Conversely, a study using avian H9N2 virus reported that loss of PA-X expression results in decreased virulence in mice [121]. While this study disagrees on the effect PA-X has on pathogenicity in vivo, there is nevertheless a clear role for PA-X in the downregulation of host immune responses during infections in both mammalian and avian hosts. As previously discussed, it has been shown that the strength of PA-X shutoff activity varies greatly between avian and mammalian IAVs raising the possibility that variations in PA-X’s effect on pathogenicity may correlate with its shutoff activities [103]. Furthermore, functional interplay and coordination between PA-X and NS1 also likely affect overall viral pathogenicity and replication in vivo [102]. The multi-functionality of NS1 as well as the varied activity of PA-X could correspond to differences in host species, with NS1 and PA-X activity fluctuating based on the presence of species-specific cellular factors. Transcriptomic analysis of infected cells from our lab found that shutoff active NS1 specifically targets cellular mRNAs related to IFN signaling pathways and cytokine release, while PA-X broadly targets cellular mRNAs [102]. These differences in targeted genes also likely affect the overall pathogenicity and replication of IAVs in distinct host species.

## 4. Clinical Interventions Targeting PA

Recently, PA has become an attractive target for therapeutic interventions against IAV because of its essential role in viral transcription and replication. Thanks in part to the large amount of structural information on PA and vRdRp, it has become more feasible to target PA with drugs and inhibitors. One such inhibitor, baloxavir marboxil (Xofluza^TM^), specifically targets the endonuclease activity of PA by binding in the endonuclease active site and inhibits replication of both influenza A and B viruses [122]. This targeting to the endonuclease active site indicates that baloxavir likely would be effective in inhibiting PA-X shutoff activity as well, although this has not been demonstrated. Baloxavir is currently licensed for usage to treat uncomplicated influenza A and B infections in both the United States and Japan. In clinical trials, baloxavir was shown to reduce viral loads and symptoms of influenza infection similar to treatment with oseltamivir (Tamiflu^TM^), a neuraminidase inhibitor [123]. However, resistance mutations in PA (I38T/F/M) have been observed to develop quickly in vitro. The mutation from isoleucine to either threonine, phenylalanine, or methionine at residue 38 is predicted to prevent baloxavir from binding in the endonuclease active site [124,125]. While targeting the vRdRp and PA/PA-X activity is promising as a treatment for acute IAV infection, these resistance mutations have been observed to arise rapidly in the human population as well. During a phase III clinical trial, 9.7% of baloxavir recipients were found to be harboring a strain with PA I38T/F/M resistance mutations after baloxavir treatment for only five days [122]. Further understanding of the IAV replication and transcription processes, as well as PA-X activity, will be helpful in designing clinical interventions that will be less prone to escape mutations.

## 5. Conclusions and Future Perspectives

While PB2 has traditionally been viewed as the major determinant for restriction of avian vRdRp activity in mammalian cells, recent studies on the H1N1pdm09 virus and avian IAVs, such as H5N1 and H7N9, found that PA plays an important role too. It is notable that many of the observed host adaptive mutations in PA are located within the endonuclease domain, which plays a critical role in the viral life cycle during both viral mRNA transcription and PA-X shutoff activity. Other host adaptive mutations are also found in surface-exposed residues on the PA C-terminal domain, which likely alter vRdRp activity through interactions with cellular factors. Overall, the multiple contributions of single mutations across various regions of PA to enhance vRdRp activity, replication, and pathogenicity in mammalian cells suggest that multiple mechanisms likely exist to modulate host adaptation. In addition to PA, the PA-X protein which is expressed from the PA gene segment has a significant impact on host gene expression and viral pathogenicity. Interestingly, PA-X proteins derived from avian IAVs appear to have greater host shutoff activity than PA-X proteins from human IAVs. The ability of PA-X to inhibit innate immune responses, such as cytokine and interferon production, is an important factor in regulating IAV virulence to facilitate efficient transmission and maintenance of the virus in the human population. Thus, the data support that there is likely modulation of PA-X activity as well during host adaptation of avian IAVs to humans and may prove crucial for the emergence of human IAVs. These facts clearly highlight the importance of PA gene products in host adaptation and clinical outcomes. In conclusion, host adaptive mutations in PA that regulate vRdRp activity and fine-tuning of PA-X activity may prove to be important factors in the emergence of new seasonal and/or pandemic IAVs. Further mechanistic studies are essential to understand how we may control and prepare for future IAV outbreaks as well as develop effective vaccines. In addition, continuing surveillance of avian IAVs, such as the H5N1 and H7N9 strains that have the capacity to infect humans, is crucial in order to screen for and identify novel host adaptive mutations. 

## Figures and Tables

**Table 1:** List of experimentally verified host adaptive avian to mammalian mutations in PA.

Mutation	Location	Strain Tested	In Vitro Effects	In Vivo Effects ^1^	Reference
A36T	Endonuclease Domain	H1N1pdm09	Enhanced vRdRp activity ^2^ and viral replication ^3^	Minimal effect on virulence	[22]
S37A	Endonuclease Domain	H7N9 (Avian)	Enhanced vRdRp activity and viral replication ^3^	No effect on virulence or replication	[66]
V63I	Endonuclease Domain	H7N7 (Avian)	Enhanced vRdRp activity ^2^ and viral replication ^4^ Enhanced endonuclease activity	Enhanced virulence	[67]
T85I	Endonuclease Domain	H1N1pdm09	Enhanced vRdRp activity ^2^, viral replication, and protein synthesis ^3^	No effect on virulence or replication	[20]
T97I	Endonuclease Domain	H5N2 (Avian)	Enhanced vRdRp activity ^2^	Enhanced replication and virulence	[68]
G186S	Endonuclease Domain	H1N1pdm09	Enhanced vRdRp activity ^2^	No effect on virulence or replication	[20]
L336M	C-Terminal Domain	H1N1pdm09	Enhanced vRdRp activity ^2^ and protein synthesis ^3^	Enhanced virulence	[20]
A343T	C-Terminal Domain	H5N1 (Avian)	Enhanced vRdRp activity ^2^ and viral replication ^3^	No effect on virulence or replication	[48]
E349G	C-Terminal Domain	H1N1pdm09	Enhanced vRdRp activity ^2^	Not tested	[69]
K356R	C-Terminal Domain	H9N2 (Avian)	Enhanced vRdRp activity ^2^, and viral replication ^3,4^	Enhanced replication and virulence	[70]
N383D	C-Terminal Domain	H5N1 (Avian)	Enhanced vRdRp activity ^2^	No effect on virulence or replication	[24]
N409S	C-Terminal Domain	H7N9 (Avian)	Enhanced vRdRp activity and viral replication ^3^	Enhanced virulence	[66]
T552S	C-Terminal Domain	H1N1pdm09	Enhanced vRdRp activity ^2^ and viral replication ^3^	Not tested	[21]

^1^ Mouse studies; ^2^ HEK 293T cells; ^3^ A549 cells; ^4^ MDCK cells. vRdRp: viral RNA-dependent RNA polymerase.
